# Community characteristics of autotrophic CO_2_-fixing bacteria in karst wetland groundwaters with different nitrogen levels

**DOI:** 10.3389/fmicb.2022.949208

**Published:** 2022-08-15

**Authors:** Xiayu Wang, Wei Li, Aoqi Cheng, Taiming Shen, Yutian Xiao, Min Zhu, Xiaodong Pan, Longjiang Yu

**Affiliations:** ^1^Institute of Resource Biology and Biotechnology, Department of Biotechnology, College of Life Science and Technology, Huazhong University of Science and Technology, Wuhan, China; ^2^College of Environmental Science and Engineering, Guilin University of Technology, Guilin, China; ^3^Key Laboratory of Karst Dynamics, MNR & GZAR, Institute of Karst Geology, Chinese Academy of Geological Sciences, Guilin, China; ^4^Key Laboratory of Molecular Biophysics, Ministry of Education, Wuhan, China

**Keywords:** CO_2_-fixing bacterial community, *cbbL* gene, *cbbM* gene, karst groundwater, nitrogen pollution

## Abstract

Karst wetlands are important in the global carbon and nitrogen cycles as well as in security of water resources. Huixian wetland (Guilin) is the largest natural karst wetland in China. In recent years, groundwater nitrogen pollution has increasingly affected the wetland ecosystem integrity due to anthropogenic activities. In this study, it was hypothesized that autotrophic microbial diversity is impacted with the advent of pollution, adversely affecting autotrophs in the carbon and nitrogen cycles. Autotrophic microbes have important roles in abating groundwater nitrogen pollution. Thus, it is of great significance to study the characteristics of autotrophic bacterial communities and their responses to environmental parameters in nitrogen-polluted karst groundwaters. The abundances of the Calvin–Benson cycle functional genes *cbbL* and *cbbM* as well as the autotrophic CO_2_-fixing bacterial communities were characterized in the karst groundwater samples with different levels of nitrogen pollution. The *cbbM* gene was generally more abundant than the *cbbL* gene in the groundwater samples. The *cbbL* gene abundance was significantly positively correlated with dissolved inorganic nitrogen (DIN) concentration (*P* < 0.01). In the autotrophic CO_2_-fixing bacterial communities, Alphaproteobacteria, Betaproteobacteria, and Gammaproteobacteria of the phylum Proteobacteria were predominant. At the genus level, *Rubrivivax* and *Methylibium* were the dominant *cbbL* gene containing genera, while *Halothiobacillus* and *Endothiovibrio* were the dominant genera for the *cbbM* gene. The abundance of autotrophic CO_2_-fixing bacterial communities increased but their diversity decreased with the inflow of nitrogen into the karst groundwater system. The community structure of autotrophic CO_2_-fixing bacteria in the groundwaters was also significantly affected by environmental factors such as the carbonic anhydrase (CA) activity, dissolved inorganic carbon (DIC) concentration, temperature, and oxidation-reduction potential (ORP). Nitrogen inflow significantly changed the characteristics of autotrophic CO_2_-fixing bacterial communities in the karst groundwaters. Some key genera such as *Nitrosospira* and *Thiobacillus* were clearly abundant in the karst groundwaters with high nitrogen levels. Their respective roles in nitrification and denitrification impact nitrogen removal in this ecosystem. The findings in this study provide an important reference for biological abatement of nitrogen pollution in the karst groundwater system.

## Introduction

The karst area of China accounts for 15.6% of the total karst area in the world and about one third of the national land area (Yuan and Cao, 2008). Karst groundwater in China accounts for more than one fourth of the total groundwater resources and is an important source of potable water and production water in karst areas (Yuan et al., 1993). These groundwaters are liable to be contaminated because of the special structures of the surface and subsurface double layers in karst areas. Sewage and agricultural non-point source pollution caused by fertilization and breeding are common sources of groundwater nitrogen pollution (Guo et al., 2006). Groundwater nitrogen pollution is a serious threat to water security. It is important, therefore, to solve problems of karst groundwater nitrogen pollution in endeavors to protect groundwater resources.

Autotrophic microbes play an important role in the treatment of groundwater nitrogen pollution (Li et al., 2020). The bedrock composition and dissolved inorganic carbon (DIC) produced by the unique water-rock-gas interaction in karst groundwater can provide the energy and carbon sources for autotrophs. Therefore, karst groundwater is a compatible environment for the growth of autotrophs. Autotrophic CO_2_-fixing microbes can assimilate CO_2_ as the only carbon source, and light or reduced inorganic compounds such as H_2_, H_2_S, S^0^, S_2_O_3_^2–^, NH_4_^+^, NO_2_^–^, or Fe^2+^ as the energy source (Yuan et al., 2011). Therefore, the autotrophic CO_2_-fixing microbes play an important role in the carbon and nitrogen cycles in aquatic ecosystems. Some autotrophs can oxidize ammonia and nitrite or reduce nitrate to N_2_ gas and can play important roles in nitrogen removal from water bodies (Wang et al., 2020). Since autotrophs do not need an organic carbon source (Jakus et al., 2021), they have potential application prospects for nitrogen removal in freshwater and groundwater habitats including karst groundwater systems.

Currently, there are six major CO_2_-fixing pathways in autotrophs: the Calvin–Benson cycle, reductive tricarboxylic acid cycle, reductive acetyl-CoA, 3-hydroxypropionate bicycle, 3-hydroxypropionate/4-hydroxybutyrate cycle, and dicarboxylate/4-hydroxybutyrate cycle (Thauer, 2007; Berg, 2011; Hügler and Sievert, 2011; Jeoung et al., 2014). The Calvin-Benson cycle is the most important pathway for bacterial assimilation of CO_2_ (Berg, 2011). The key enzyme is the ribulose 1,5-bisphosphate carboxylase/oxygenase (RubisCO) that catalyzes the carboxylation of ribulose 1,5-bisphosphate to yield two molecules of 3-phosphoglycerate (Hügler and Sievert, 2011). The RubisCO Type I (encoded by gene *cbbL*) and Type II (encoded by gene *cbbM*) are the main and most studied RubisCO enzymes (Berg, 2011; Liu et al., 2017). Due to the high conservation of these two functional genes and the appropriate nucleotide length, *cbbL* and *cbbM* genes have been used as specific probes for molecular analysis of the autotrophic microbial communities in the environment (Wu et al., 2014; Wang et al., 2021). These genes have also been used to characterize the diversity of autotrophic bacteria and their responses to environmental conditions (Long et al., 2015; Wrighton et al., 2016; Alfreider et al., 2017). However, there are only few karst groundwater studies on the characterization of autotrophic microbial communities and their responses to environmental changes.

The Huixian wetland in Guilin, Guangxi Province is the most representative karst wetland in China and even in the global tropical, subtropical middle, and low altitude karst areas (Cai et al., 2012). It is the largest natural karst wetland of China. The groundwater in the Huixian karst wetland was affected by sewage pollution and non-point source pollution caused mostly by farming and agriculture (Peng et al., 2018). It was hypothesized that autotrophic microbial diversity is impacted with the advent of pollution, adversely affecting autotrophs in the carbon and nitrogen cycles of karst wetland system. The purpose of this study was to characterize the autotrophic CO_2_-fixing bacterial communities in the Huixian karst groundwaters with different nitrogen levels and their relationships with dissolved inorganic nitrogen species and other selected groundwater environmental factors. A karst groundwater vein with different nitrogen levels in the aquifer was selected in the Huixian karst wetland for this study. Of special interest were the differences in the community structures of CO_2_-fixing bacteria in the karst groundwaters with high and low nitrogen levels because these differences are poorly understood in karst groundwaters. The results of this study will provide a scientific and biological basis for removal of nitrogen pollution, and an important reference for the protection and utilization of karst groundwater resources.

## Materials and methods

### Study area

The study area is in the Huixian karst wetland, Fengjia Village, Huixian County, Guilin, Guangxi Province, China. The Huixian karst wetland belongs to the subtropical monsoon climate with an annual average temperature range of 16.5∼20.5°C. The temperature in the summer is significantly different from that in the winter. The temperature difference between the spring and autumn is small, and the temperature in the autumn is slightly higher than that in the spring. The average annual rainfall is about 1890.4 mm. The rainy season is mainly concentrated from April to July, and October through March next year is the dry season, with January being the driest and coldest period (Cai et al., 2012).

### Sampling

**FIGURE 1:**
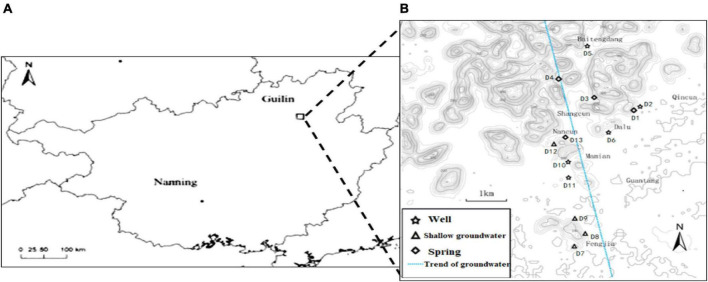
Schematic diagram of the study area and sampling sites in the Huixian karst groundwaters. **(A)** Location of the Huixian karst wetland; **(B)** Location of the sampling sites in the Huixian karst groundwaters.

A groundwater vein (110°13′31″ ∼ 110°15′10″E, 25°09′13″ ∼ 25°05′51″N) from Baitengdang in the north to Fengjia in the south was selected in the east of Huixian karst wetland, as shown in Figure 1. Villages and farmlands are widely distributed along this groundwater vein. The main villages it flows through are Baitengdang, Qin Village, Shang Village, Nan Village, Dalu Village, Mamian Village and Fengjia Village, of which Fengjia Village has a large area of farming and agriculture. Thirteen groundwater sampling sites (D1–D13) were selected from the north to south transect along this groundwater vein. They represented three types of karst groundwater, namely a karst spring (Spring), a shallow pumping well (Well), and a shallow underground river (Shallow groundwater). Sites D1, D3, D4, and D13 were karst springs, sites D2, D5, D6, D10, and D11 were shallow pumping wells, and sites D7, D8, D9, and D12 were shallow underground rivers. The lithology of the sampling sites was mainly limestone, and mixed limestone and dolomite. The land use patterns around the sampling sites were mainly shrubs and farmland. The hydrogeological information and land use patterns of various sampling sites are presented in Table 1.

**TABLE 1:** Hydrogeological information and land use patterns at various sampling sites in the Huixian karst groundwaters, China.

Site no.	Groundwater type	Lithology	Land use pattern
D1	Karst spring	Limestone	Shrub, farmland
D2	Shallow pumping well	Limestone	Shrub
D3	Karst spring	Dolomite and limestone mixed lithology	Shrub, woodland
D4	Karst spring	Dolomite and limestone mixed lithology	Shrub
D5	Shallow pumping well	Dolomite and limestone mixed lithology	Abandoned farmland
D6	Shallow pumping well	Limestone	Shrub, farmland
D7	Shallow underground river	Limestone	Woodland
D8	Shallow underground river	Limestone	Farmland
D9	Shallow underground river	Limestone	Farmland
D10	Shallow pumping well	Limestone	Farmland, breeding farm
D11	Shallow pumping well	Limestone	Farmland, breeding farm
D12	Shallow underground river	Limestone	Shrub, farmland
D13	Karst spring	Limestone	Shrub, woodland

Sampling was carried out in January (winter, 201801), April (spring, 201804), July (summer, 201807), and October (autumn, 201810). Before sampling, the sampler (a polyethylene plastic bucket) was first washed three times with purified water followed by three rinses with groundwater from the sampling site. At each site, 5–10 L groundwater samples were collected from three points, then combined and mixed, and stored in polyethylene plastic containers. The samples were filtered with 0.22 μm cellulose acetate membrane filters to entrap the microorganisms from the groundwater. The membrane filters were then placed in sterile petri dishes and then placed in a dry-ice box, brought back to the laboratory and stored at –80°C for total DNA extraction.

About 500 mL of filtered groundwater samples were acidified with 2 mL of 1 mol/L sulfuric acid and stored in 500 mL polyethylene bottles in an ice box, brought back to the laboratory and stored at 4°C for analysis of NH_4_^+^, NO_2_^–^, NO_3_^–^, SO_4_^2–^, and dissolved organic carbon (DOC). For the analysis of total nitrogen (TN) and total organic carbon (TOC), about 500 mL unfiltered groundwater samples at each site were fixed with 2 mL of saturated HgCl_2_ solution, stored in 500 mL polyethylene bottles in an ice box, brought back to the laboratory, and stored at 4°C before analysis.

### Determination of groundwater physicochemical properties

Groundwater physicochemical parameters, including pH, temperature (T), turbidity, conductivity, oxidation-reduction potential (ORP), dissolved oxygen (DO), Ca^2+^, and DIC (HCO_3_^–^) concentration were measured *in situ*. The pH was measured using a Mettler-Toledo Seven2GO S2 portable pH meter with an accuracy of ± 0.01 pH unit. The T and turbidity were measured using a Eureka Manta2 multi-parameter water quality monitor (Eureka Water Probes, Austin, TX, United States). The accuracy of the measurements was ±0.1°C and ±0.02 NTU, respectively. The conductivity was measured using a CT-3030 conductivity probe (Kedida, Shenzhen) with an accuracy of ± 0.01 mS/cm. The ORP was measured using a CT-8022 portable ORP pen (Kedida, Shenzhen) with an accuracy of ±0.1 mV. The DO was measured using a JPB-607A portable dissolved oxygen meter (Ramag, Shanghai) with an accuracy of ± 0.01 mg /L. The concentration of Ca^2+^ was measured using a Aquamerck Calcium Kit (Merck) with an accuracy of ±0.1 mg/L. The concentration of DIC was measured using an Aquamerck Alkalinity Kit (Merck) with an accuracy of ±0.05 mmol/L (Nzung’a et al., 2018).

The concentrations of TN, NH_4_^+^, NO_2_^–^, and NO_3_^–^ of groundwater samples were determined according to the Methods for Monitoring and Analyzing Water and Waste water (2002). The concentration of dissolved inorganic nitrogen (DIN) is the sum of NH_4_^+^, NO_2_^–^, and NO_3_^–^ concentrations. The TOC and DOC concentrations were determined using a total organic carbon analyzer (Multi N/C 2100, Analytik, Jena, Germany). The SO_4_^2–^ concentration was determined using an Ion Chromatography with an accuracy of ± 0.01 mg/L.

### Measurement of groundwater carbonic anhydrase activity

The carbonic anhydrase (CA) activity of groundwater samples was determined from the rate of CO_2_ hydration by following the change of pH traced on a chart recorder as previously described (Li et al., 2005; Nzung’a et al., 2018). The basic physicochemical properties and CA activity at various sites of the Huixian karst groundwaters are presented in Table 2.

**TABLE 2:** Basic physicochemical properties and CA activity at the sampling sites of the Huixian karst groundwater.

Site No.	T (°C)	pH	DO (mg/L)	ORP (mV)	Conductivity (mS/cm)	Turbidity (NTU)	Ca^2+^ (mg/L)	SO_4_^2–^ (mg/L)	CA (U/mL)
D1	20.5 ± 3.67	7.38 ± 0.24	4.23 ± 1.06	262.75 ± 23.68	0.67 ± 0.04	6.7 ± 4.58	110.88 ± 21.93	33.08 ± 30.4	0.77 ± 0.12
D2	20.45 ± 6.08	7.48 ± 0.15	6.53 ± 0.48	248.75 ± 19.47	0.59 ± 0.08	13.98 ± 14.73	106.25 ± 11.03	11.84 ± 5.57	0.8 ± 0.11
D3	18.75 ± 1.6	7.38 ± 0.03	6.75 ± 0.25	263.25 ± 30.41	0.62 ± 0.1	4.8 ± 2.57	115.25 ± 14.22	10.46 ± 3.35	1.01 ± 0.23
D4	19.83 ± 2.35	7.16 ± 0.11	6.25 ± 0.66	253.75 ± 17.39	0.69 ± 0.15	3.75 ± 3.1	114.25 ± 18.59	22.96 ± 12.53	0.85 ± 0.14
D5	22.69 ± 4.31	7.54 ± 0.34	6.05 ± 0.91	252.75 ± 23.37	0.54 ± 0.07	7.05 ± 6.01	101.25 ± 13.45	15.39 ± 7.72	0.83 ± 0.09
D6	19.9 ± 4.1	7.4 ± 0.3	5.33 ± 1.65	270.5 ± 33.31	0.86 ± 0.08	1.65 ± 2.04	149.3 ± 4.25	117.17 ± 40.57	0.71 ± 0.17
D7	20.6 ± 6.3	7.33 ± 0.08	4.18 ± 1.92	249.75 ± 26.94	0.52 ± 0.08	18.68 ± 30.13	84 ± 12.19	18.22 ± 18.54	0.55 ± 0.13
D8	20.34 ± 5.05	7.46 ± 0.09	5.65 ± 0.99	250.75 ± 29.3	0.55 ± 0.06	4.3 ± 2.43	99.75 ± 11.67	15.44 ± 13.36	0.71 ± 0.14
D9	22.34 ± 6.36	7.87 ± 0.3	5.9 ± 1.49	248.5 ± 35.87	0.59 ± 0.16	11.48 ± 3.43	98.5 ± 32.72	36.8 ± 52.48	0.69 ± 0.15
D10	21.02 ± 0.97	7.31 ± 0.16	4.3 ± 0.88	256.25 ± 25.57	0.6 ± 0.08	15.38 ± 27.44	101.13 ± 6.38	23.19 ± 18.9	0.63 ± 0.14
D11	21.95 ± 1.31	7.3 ± 0.13	4.88 ± 0.85	252.5 ± 31.42	0.53 ± 0.06	3.13 ± 2.2	85.38 ± 12.05	11.72 ± 20.16	0.48 ± 0.04
D12	20.17 ± 2.98	7.27 ± 0.07	4.48 ± 1.11	256.25 ± 28.49	0.63 ± 0.09	6.5 ± 4.14	114.25 ± 6.18	28.98 ± 22.91	0.82 ± 0.07
D13	18.96 ± 1.56	7.34 ± 0.18	5.75 ± 0.57	254.75 ± 36.63	0.66 ± 0.12	3.75 ± 3.61	114.38 ± 2.93	26.85 ± 22	0.86 ± 0.15

**Site No.**	**TN** **(mg/L)**	**NH_4_^+^** **(mg/L)**	**NO_2_^–^** **(mg/L)**	**NO_3_^–^** **(mg/L)**	**DIN** **(mg/L)**	**TOC** **(mg/L)**	**DOC** **(mg/L)**	**DIC** **(mmol/L)**	

D1	17.15 ± 9.71	3.48 ± 6.55	0.31 ± 0.52	1.79 ± 1.42	5.58 ± 7.52	2.9 ± 1.13	1.13 ± 1.53	4.81 ± 0.57	
D2	16.45 ± 7.46	0.21 ± 0.15	0.02 ± 0.02	0.95 ± 0.36	1.19 ± 0.31	1.26 ± 0.82	0.51 ± 1.02	5.05 ± 0.37	
D3	17.58 ± 5.57	1.28 ± 2.21	0.02 ± 0.03	0.48 ± 0.18	1.78 ± 2.13	3.1 ± 2.08	0.55 ± 1.02	5.44 ± 0.77	
D4	17.59 ± 6.52	0.24 ± 0.16	0.04 ± 0.06	0.43 ± 0.21	0.70 ± 0.30	2.16 ± 2.49	0.51 ± 0.83	5.49 ± 0.96	
D5	16.5 ± 8.15	0.2 ± 0.14	0.03 ± 0.03	0.68 ± 0.44	0.91 ± 0.55	0.93 ± 0.86	0.46 ± 0.63	4.64 ± 0.88	
D6	18.62 ± 8.2	0.24 ± 0.14	0.02 ± 0.01	7.05 ± 2.53	7.32 ± 2.67	1.25 ± 2.05	0.64 ± 1.29	4.56 ± 0.39	
D7	16.46 ± 4.53	1.3 ± 2.2	0.31 ± 0.43	1.2 ± 0.51	2.81 ± 2.54	4.53 ± 3.09	1.6 ± 0.54	3.7 ± 0.86	
D8	16.96 ± 7.7	0.21 ± 0.16	0.02 ± 0.03	3.43 ± 2.74	3.66 ± 2.80	5.08 ± 4.22	1.04 ± 1.11	4.24 ± 0.38	
D9	15.31 ± 5.34	9.74 ± 9.19	0.32 ± 0.29	1.7 ± 1.52	11.77 ± 9.22	9.64 ± 11.74	1.85 ± 1.42	4.29 ± 0.9	
D10	18.78 ± 8.07	0.2 ± 0.15	0.02 ± 0.02	8.62 ± 1.23	8.84 ± 1.15	1.67 ± 1.59	0.65 ± 1.31	4.08 ± 0.53	
D11	18.98 ± 6.85	0.21 ± 0.14	0.09 ± 0.13	10.9 ± 5.61	11.19 ± 5.70	1.39 ± 1.85	0.61 ± 1.22	2.68 ± 0.82	
D12	15.94 ± 6.15	0.19 ± 0.13	0.06 ± 0.1	2.51 ± 2.51	2.75 ± 2.67	6.04 ± 8.03	0.75 ± 0.87	5.18 ± 0.19	
D13	20.08 ± 3.87	0.19 ± 0.14	0.03 ± 0.05	1.76 ± 1.39	1.98 ± 1.44	2.14 ± 1.52	0.72 ± 1.17	5.24 ± 0.15	

The data are the average value ± standard deviation of the groundwater samples measured in January, April, July, and October 2018.

### Extraction of total DNA of groundwater microorganisms

PowerWater DNA Isolation Kit (Qiagen, Hilden, Germany) were used to extract the total DNA of the biomass on the membrane filters according to the instruction of the manufacturer. The DNA was dissolved in 100 μL of sterile deionized water, and the quality was checked by 1% agarose gel electrophoresis. DNA concentration and purity were determined with a NanoDrop ND-1000 spectrophotometer (Thermo Fisher Scientific, Waltham, MA, United States). The DNA samples were stored at –80°C for subsequent analysis.

### Detection of *cbbL* and *cbbM* genes

The abundances of the 16S rRNA gene and the *cbbL* and *cbbM* genes of bacteria in the Huixian karst groundwater samples were analyzed by quantitative PCR (qPCR). The amplification primers are shown in Supplementary Table 1. The reaction mixture was 10 μL including 5 μL SYBR PremixEX TaqTM, 0.5 μL 10 mM forward primer, 0.5 μL 10 mM reverse primer, 1 μL template DNA, and 3 μL ddH_2_O. The reaction conditions were as follows: pre-denaturation at 95°C for 60 s; 40 cycles of denaturation at 95°C for 10 s, annealing at specific annealing temperature for 10 s, and extension at 72°C for 30 s; and melting at 95°C for 15 s, 60°C for 60 s, and 95°C for 15 s. The annealing temperatures for the 16S rRNA, *cbbL*, and *cbbM* genes were 50, 54, and 57°C, respectively.

### High-throughput sequencing

The corresponding primers listed in Supplementary Table 1 were used to amplify the *cbbL* and *cbbM* genes by PCR. The PCR reaction system was 30 μL including 3 μL template DNA, 25 μL 2 × Premix Taq, 1 μL 10 mM forward primer, and 1 μL 10 mM reverse primer. The reaction conditions were as follows: pre-denaturation at 94°C for 5 min; 30 cycles of denaturation at 94°C for 30 s, annealing at 54°C (*cbbL*) or 57°C (*cbbM*) for 30 s, extension at 72°C for 30 s, and a final extension at 72°C for 10 min. The PCR amplification products were detected by 2% agarose gel electrophoresis, and the target fragments were cut and recovered using a gel recovery kit (Omega Bio-tek, Norcross, GA, United States). The recovered target gene fragments were sequenced on an Illumina MiSeq 300 platform by Personal Biotechnology Co., Ltd. (Shanghai, China).

### Sequence analysis

The raw data obtained by paired-end sequencing were first spliced by FLASH and then filtered by the Quantitative Insights Into Microbial Ecology (QIIME, v1.8.0) pipeline (Caporaso et al., 2010). After removing low-quality bases and adaptor contamination sequences, the data filtering was completed. The chimeras were removed by UCHIME software to obtain effective sequences. The effective sequences were clustered into operational taxonomic units (OTUs) at 97% sequence similarity using the UCLUST method in QIIME software (Edgar, 2010). The Alpha diversity indexes (Chao 1, ACE, Shannon, and Simpson indexes) of the samples were calculated by QIIME software, and the OTU taxonomic classification was conducted by BLAST searching the representative sequences set against the NCBI NT Database using the best hit.

The raw sequences of CO_2_-fixing bacteria in the Huixian karst groundwaters were deposited in the NCBI Sequence Read Archive under the accession number PRJNA821389.

### Statistical analysis

Differences in the abundances of the *cbbL* and *cbbM* genes among different seasons were analyzed by one-way analysis of variance (ANOVA) at *P* < 0.05 using SPSS 18.0. Relationships between the groundwater environmental parameters and functional gene abundances as well as diversity indexes were analyzed by the Pearson correlation using SPSS 18.0. Relationships between the groundwater environmental parameters and the relative abundance of bacterial genera were analyzed by the Spearman correlation heatmap using SPSS 18.0. The redundancy analysis (RDA) was performed to explore the relationship between the CO_2_-fixing bacterial community structure and groundwater environmental parameters using the R software package. The linear discriminant analysis (LDA) effect size (LEfSe) method was used to identify significant differences in the community structure of CO_2_-fixing bacteria in the karst groundwaters between the high (HN) and low (LN) nitrogen levels using the galaxy platform^1^. Histograms of linear discriminate analysis (LDA) and cladograms of phylogenetic distribution were prepared to present the differentially abundant carbon-fixing bacterial taxa in the karst groundwaters. Variance partitioning analysis (VPA) was performed to distinguish the relative contribution of water environmental factors associated with carbon (environmental carbon factors for short) and water environmental factors associated with nitrogen (environmental nitrogen factors) on the autotrophic CO_2_-fixing bacterial communities in the karst groundwaters using the “varpart” function in the vegan package. The environmental carbon factors included DIC, DOC, TOC, and CA, and the environmental nitrogen factors included DIN, TN, NH_4_^+^, NO_2_^–^, and NO_3_^–^.

## Results

### Variation of carbon- fixing genes abundance in the karst groundwaters

**FIGURE 2:**
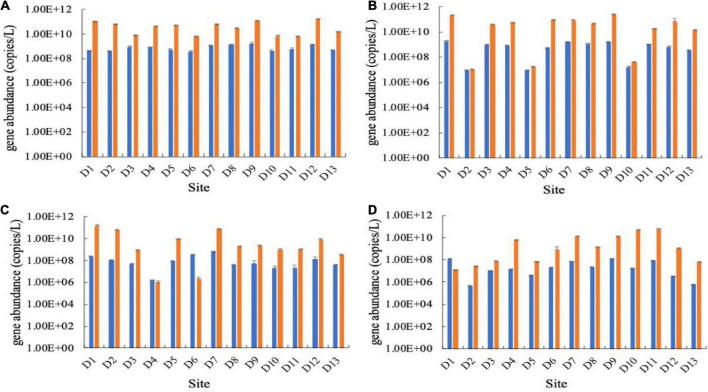
Abundances of the *cbbL* (left, blue) and *cbbM* (right, orange) genes in the Huixian karst groundwaters. **(A)** Winter (201801); **(B)** Spring (201804); **(C)** Summer (201807); **(D)** Autumn (201810).

In the Huixian karst groundwaters, the abundance of the carbon-fixing gene *cbbM* was generally higher than that of *cbbL*. The abundance of the *cbbM* gene in all samples ranged from 9.76 × 10^5^ to 2.56 × 10^11^ copies/L, while the *cbbL* gene ranged from 4.93 × 10^5^ to 1.79 × 10^9^ copies/L in four seasons (Figure 2). The abundances of the *cbbL* and *cbbM* genes were significantly different among the four seasons (*P <* 0.05), and they were also significantly higher in the winter and spring than in the summer and autumn (*P <* 0.05).

**FIGURE 3:**
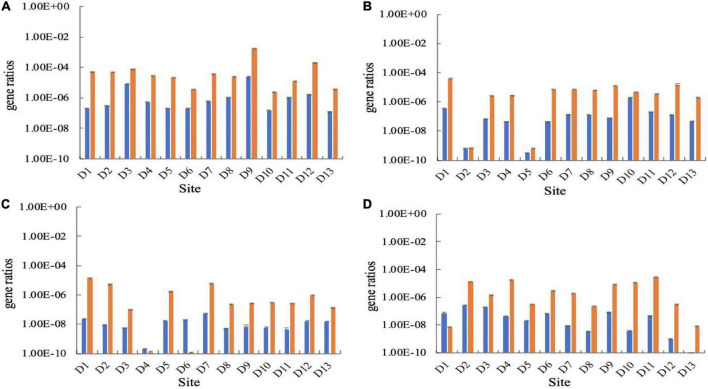
Ratios of gene copy numbers of *cbbL* (left, blue) or *cbbM* (right, orange) to 16S rRNA genes in the Huixian karst groundwaters. **(A)** Winter (201801); **(B)** Spring (201804); **(C)** Summer (201807); **(D)** Autumn (201810).

The proportion of potential CO_2_-fixing bacteria in the bacterial community in the karst groundwaters was estimated based on the abundances of the 16S rRNA, *cbbL* and *cbbM* genes. In all samples, the ratio of *cbbL*/16S rRNA gene ranged from 9.68 × 10^–11^ to 2.31 × 10^–5^, while the ratio of *cbbM*/16S rRNA ranged from 1.08 × 10^–10^ to 1.75 × 10^–3^. The proportion of the *cbbL* gene was generally lower than that of *cbbM* gene except at a few sampling sites (Figure 3). The results of the one-way ANOVA showed that there was no significant difference in the proportion of carbon-fixing genes among the seasons.

### Effects of nitrogen and other environmental factors on the abundance of CO_2_-fixing genes

**FIGURE 4:**
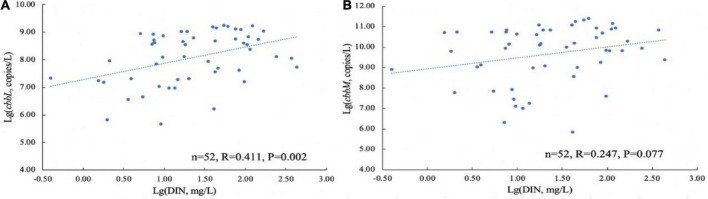
Relationship between the carbon-fixing genes and DIN concentration in the Huixian karst groundwaters. **(A)** *cbbL* gene; **(B)** *cbbM* gene.

To investigate whether nitrogen levels influenced the abundance of CO_2_-fixing bacterial community in the karst groundwaters, Pearson correlation analysis was conducted between the abundance of CO_2_-fixing genes and DIN content. The results showed that the *cbbL* gene abundance was significantly positively correlated with the DIN content (*P <* 0.01). The higher the nitrogen level, the higher the *cbbL* gene abundance (Figure 4). Although the *cbbM* gene abundance was positively correlated with DIN content, the correlation was not significant (*P* = 0.077).

The results of Pearson correlation analysis between the abundance of carbon-fixing genes and various environmental factors showed that the *cbbL* gene abundance was significantly positively correlated with ORP, DOC, and turbidity, while negatively correlated with groundwater T (*P <* 0.05). The *cbbM* gene abundance was significantly positively correlated with ORP, TOC and DOC, while negatively correlated with the CA activity (*P* < 0.05) (Table 3).

**TABLE 3:** Results of the correlation analysis between carbon-fixing gene abundances and various environmental factors in the Huixian karst groundwaters.

	*cbbL*	*cbbM*
CA	–0.230	–0.297*
TN	0.078	0.055
NH_4_^+^	0.154	0.058
NO_2_^–^	0.009	0.102
NO_3_^–^	0.141	0.070
DO	0.056	0.038
Ca^2+^	0.019	–0.071
ORP	0.524**	0.305*
DIC	–0.111	–0.111
pH	–0.069	–0.067
Conductivity	–0.124	–0.199
T	–0.342*	–0.200
Turbidity	0.345*	0.178
TOC	0.206	0.291*
DOC	0.324*	0.284*
SO_4_^2–^	–0.081	0.084

*P < 0.05; **P < 0.01.

### Community structure and diversity of the CO_2_-fixing bacteria in the karst groundwaters

The results showed that the physicochemical properties and the CO_2_-fixing gene abundances in the Huixian karst groundwaters were significantly different between summer (201807) and winter (201801) (Table 1 and Figure 2). Therefore, the summer and winter were selected as the representatives of rainy season and dry season, respectively. High-throughput sequencing analysis was carried out on the groundwater samples in these two seasons to investigate the community characteristics of CO_2_-fixing bacteria. The rarefaction curves of the *cbbL* and *cbbM* genes reached the saturation plateau (Supplementary Figure 1), suggesting that the sequencing depth basically covered all species in the samples. The observed species richness in the CO_2_-fixing bacterial community containing the *cbbL* and *cbbM* genes in all samples was within 1200. The richness (ACE, Chao1) and diversity indexes (Simpson and Shannon) of the *cbbL* gene-containing bacterial community were higher than those of the *cbbM* gene except for a few sampling sites (D1, D2, D10, and D12) in the summer (Supplementary Tables 2, 3). This indicated that the diversity of CO_2_-fixing bacterial community using RubisCO Type I was generally higher than that using RubisCO Type II for carbon assimilation in the Huixian karst groundwaters.

A total of 26 samples were obtained for sequencing from the Huixian karst groundwaters representing the summer and winter seasons. 645429 sequences and 85985 OTUs were obtained from the *cbbL* gene sequencing, belonging to 7 phyla, 11 classes, 17 orders, 30 families and 45 genera. 1111850 sequences and 114438 OTUs were obtained from the *cbbM* gene sequencing, belonging to 1 phylum, 4 classes, 8 orders, 15 families, and 23 genera.

**FIGURE 5:**
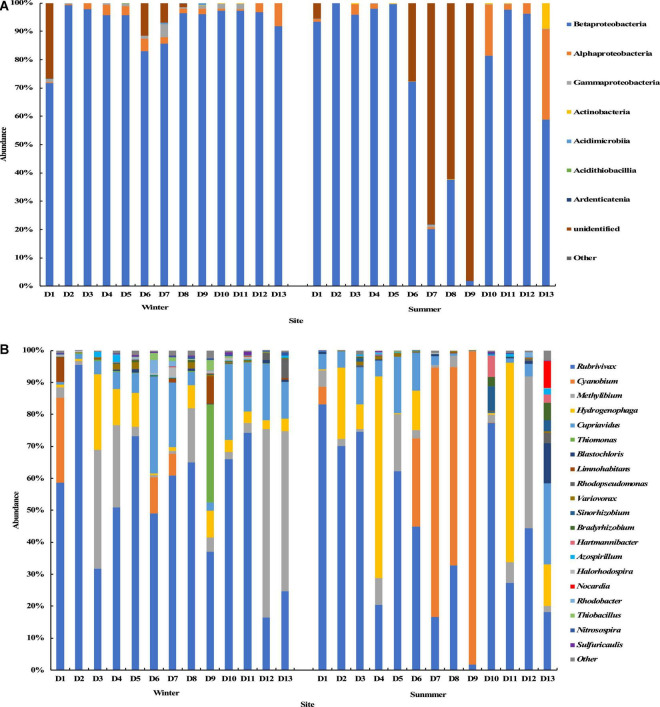
Relative abundance and composition of the *cbbL* gene-containing CO_2_-fixing bacterial community at the class level **(A)** and genus level **(B)** in the Huixian karst groundwaters.

The results of the *cbbL* gene sequencing showed that at the phylum level, Proteobacteria was dominant in the groundwater samples from most sites, and its average relative abundance was 95.97% with a maximum of 100%. In summer, the Cyanobacteria phylum was dominant with an average relative abundance of 79.48% in the water samples from sites D7, D8, and D9. At the class level, except for a few sampling sites (D7, D8, and D9) in summer, Betaproteobacteria was in the dominant position with an average relative abundance of 83% in most sampling sites, followed by Alphaproteobacteria with an average relative abundance of 3.7%. Many Cyanobacteria species could not be identified at the class level in the samples from sites D7, D8, and D9 in the summer (Figure 5A). At the genus level, the top 20 genera with the relative abundance were selected to construct the distribution graph of the relative abundance of species (Figure 5B). The top genera were *Rubrivivax*, *Cyanobium*, *Methyllium*, *Hydrogenophaga*, and *Cupriavidus* with a total relative abundance of 92.1%. *Rubrivivax* was the most abundant species in most groundwater samples, and was found in the groundwater samples from all sites in the winter and summer seasons, with the highest relative abundance of 83.1% and the average relative abundance of 49.1%. *Cyanobium* was the second and was found only in the groundwater samples from sites D1, D6 and D7 in the winter and from sites D1, D6, D7, D8, and D9 in the summer. Its relative abundance in the samples from site D9 in the summer was the highest (98.1%), and the overall average relative abundance was 12.2%. *Methyllium* was found at all sampling sites in the winter and summer seasons, with an average relative abundance of 11.8%. It was dominant at sites D12 and D13 in the winter, with the relative abundance of 59.0 and 50.2% respectively. Except for the samples from sites D7, D8, D9, and D12 in the summer, *Hydrogenophaga* was found in the samples from other sites, with an average relative abundance of 10.0%. *Cupriavidus* was found at all sampling sites in the winter and summer, with an average relative abundance of 9.0%. The relative abundance ranged from 0.1 to 30.3%.

**FIGURE 6:**
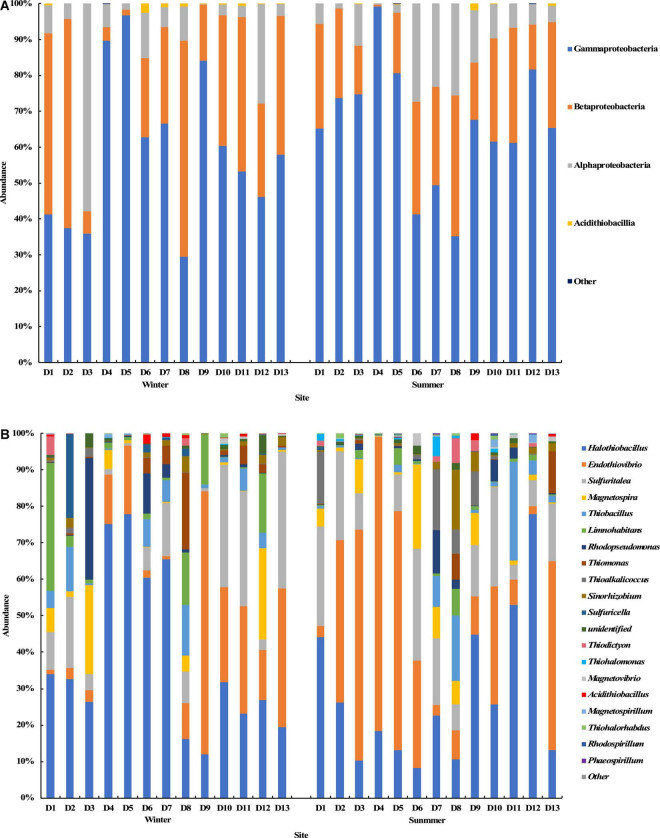
Relative abundance and composition of the *cbbM* gene-containing CO_2_-fixing bacterial community at the class level **(A)** and genus level **(B)** in the Huixian karst groundwaters.

The results of the *cbbM* gene sequencing showed that Proteobacteria was also the dominant bacterial phylum at all sampling sites in the summer and winter seasons (Figure 6). At the class level, Gammaproteobacteria, Betaproteobacteria, and Alphaproteobacteria were dominant in all groundwater samples with average relative abundances of 62.3, 26.6, and 10.8%, respectively (Figure 6A). At the genus level, the top 20 genera with relative abundance were selected to construct the distribution graph of the relative abundance of species (Figure 6B). *Halothiobacillus*, *Endothiovibrio*, *Sulfuritalea*, *Magnetospira*, *Thiobacillus*, *Limnohabitans*, and *Rhodopseudomonas* were among the abundant genera, with average relative abundances of 33.4, 24.4, 14.2, 5.2, 4.8, 4.2, and 3.0%, respectively. Their total relative abundance was up to 89.2%. *Halothiobacillus* had the highest average relative abundance and was found at all sampling sites. It was dominant at sites D4, D5, D6, and D7 in the winter and at sites D11 and D12 in the summer, and the highest relative abundance was 77.8%. *Endothiovibrio* was found at all sampling sites in the winter and summer. It was dominant at site D9 in the winter and at sites D3, D4, D5, and D13 in the summer, and its relative abundance at site D4 in the summer was the highest (80.6%). *Sulfuratalea*, *Magnetospira*, *Thiobacillus*, and *Limnohabitans* were found at all sampling sites in the winter and summer. The relative abundance of *Limnohabitans* in the groundwater samples from site D1 in winter was much higher than in other samples, and the relative abundance was 35.0%. The relative abundance of *Rhodopseudomonas* in the groundwater samples at site D3 in the winter was much higher than in other samples, and the relative abundance was 33.2%.

Thus, these results indicated that there were substantial temporal and spatial differences in the community structure of CO_2_-fixing bacteria containing the *cbbL* and *cbbM* genes in the Huixian karst groundwaters.

### Distribution of CO_2_-fixing bacterial communities as affected by dissolved inorganic nitrogen levels

**FIGURE 7:**
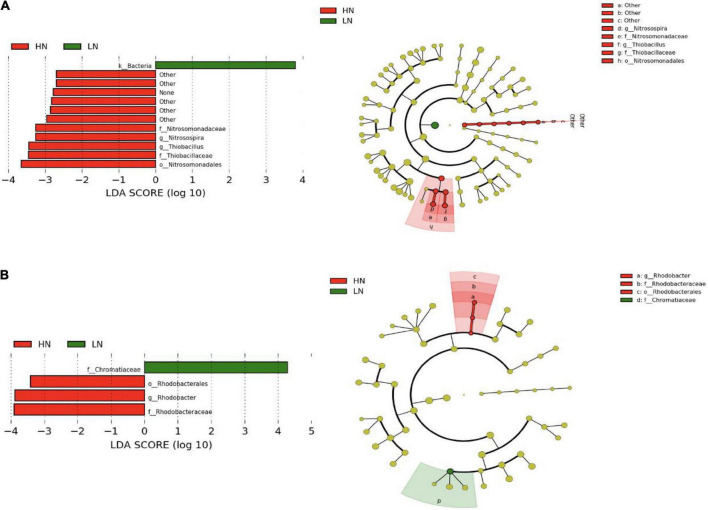
Differentially abundant CO_2_-fixing bacterial taxa as assessed using a histogram of linear discriminate analysis (LDA) and cladogram of phylogenetic distribution with effect size measurements (LEfSe) in the Huixian karst groundwaters. **(A)** *cbbL* gene; **(B)** *cbbM* gene.

According to the DIN concentration, the groundwater samples in the winter and summer were divided into high (DIN > 3.0 mg/L) and low nitrogen levels. The results of LEfSe analysis showed that there were significant differences in the distribution of some CO_2_-fixing bacterial taxa in the groundwater samples between the high and low nitrogen levels. For the *cbbL* gene-containing CO_2_-fixing bacterial communities, some autotrophs such as Nitrosomonadales, Nitrosomonadaceae, Thiobacillaceae, *Nitrosospira* and *Thiobacillus* were relatively abundant in the groundwaters with high nitrogen level (Figure 7A). For the *cbbM* gene-containing CO_2_-fixing bacterial communities, Rhodobacterales, Rhodobacteraceae and *Rhodobacter* were significantly more abundant in the groundwaters with high nitrogen level, while Chromatiaceae was significantly more abundant with low nitrogen level (Figure 7B).

### Effects of environmental factors on the structure and diversity of CO_2_-fixing bacterial community

Pearson correlation analysis was conducted between the richness indexes ACE and Chao1 as well as the diversity indexes Simpson and Shannon of the CO_2_-fixing bacterial community and environmental factors. The richness and diversity indexes of the *cbbL* gene-containing CO_2_-fixing bacterial community were significantly positively correlated with ORP in water (*P <* 0.05), while significantly negatively correlated with the concentration of inorganic nitrogen compounds in water (*P* < 0.05) (Table 4). The richness and diversity indexes of the *cbbM* gene*-*containing CO_2_-fixing bacterial community were significantly negatively correlated with NH_4_^+^, NO_2_^–^, and DO concentrations in water (*P <* 0.05) (Table 5).

**TABLE 4:** Results of the correlation analysis between the community diversity of the *cbbL* gene-containing carbon-fixing bacteria and environmental factors in the Huixian karst groundwaters.

Environmental factors	ACE	Chao1	Simpson	Shannon
CA	–0.258	–0.236	–0.148	–0.139
TN	0.185	0.208	0.262	0.167
NH_4_^+^	–0.163	–0.166	–0.18	–0.204
NO_2_^–^	–0.191	–0.194	–0.207	–0.232
NO_3_^–^	0.249	0.216	–0.153	–0.053
DO	–0.181	–0.164	–0.115	–0.156
Ca^2+^	0.286	0.297	–0.021	0.172
ORP	0.474*	0.491*	0.233*	0.389*
DIC	0.100	0.132	–0.049	0.126
pH	–0.206	–0.225	–0.081	–0.236
conductivity	0.107	0.105	–0.177	–0.03
T	–0.276	–0.302	–0.153	–0.217
turbidity	0.067	0.061	–0.228	–0.083
TOC	–0.266	–0.27	0.035	–0.208
DOC	0.267	0.265	0.265	0.247
SO_4_^2–^	0.295	0.26	–0.036	0.022
DIN	–0.406*	–0.443*	–0.449*	–0.513**

*P < 0.05; **P < 0.01.

**TABLE 5:** Results of the correlation analysis between the community diversity of the *cbbM* gene-containing carbon-fixing bacteria and environmental factors in the Huixian karst groundwaters.

Environmental factors	ACE	Chao1	Simpson	Shannon
CA	–0.012	0.004	–0.086	–0.103
TN	–0.201	–0.216	–0.274	–0.335
NH_4_^+^	–0.383	–0.399*	–0.720**	–0.646**
NO_2_–	–0.377	–0.392*	–0.705**	–0.635**
NO_3_–	0.446*	0.438	0.136	0.228
DO	–0.404*	–0.427*	–0.518**	–0.594**
Ca^2+^	–0.109	–0.104	–0.303	–0.304
ORP	0.062	0.064	–0.030	0.015
DIC	–0.002	0.001	–0.306	–0.267
pH	–0.339	–0.367	–0.272	–0.300
conductivity	0.012	0.027	–0.159	–0.154
T	0.184	0.189	0.291	0.341
turbidity	0.009	–0.001	0.031	0.023
TOC	–0.146	–0.161	0.104	0.065
DOC	–0.194	–0.217	–0.153	–0.131
SO_4_^2^–	0.343	0.318	0.145	0.236
DIN	0.159	0.137	0.232	0.246

*P < 0.05; **P < 0.01.

**FIGURE 8:**
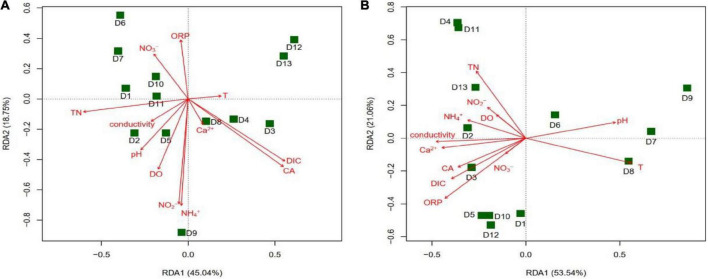
Redundancy analysis (RDA) of the *cbbL* gene-containing CO_2_-fixing bacterial community structures with environmental factors in the Huixian karst groundwaters. **(A)** Winter (201801); **(B)** Summer (201807).

**FIGURE 9:**
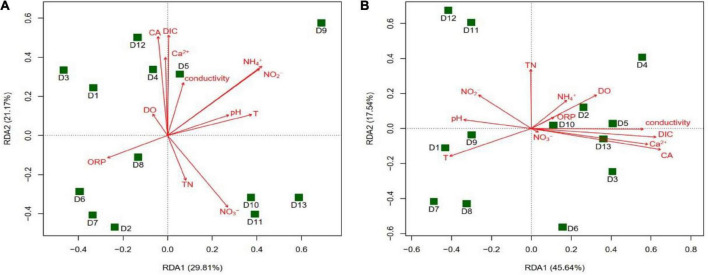
Redundancy analysis (RDA) of the *cbbM* gene-containing CO_2_-fixing bacterial community structures with environmental factors in the Huixian karst groundwaters. **(A)** Winter (201801); **(B)** Summer (201807).

The results of the RDA analysis showed that the main groundwater environmental factors affecting the community structure of the *cbbL* gene-containing CO_2_-fixing bacteria in the winter were CA (*r*^2^ = 0.5270, *P* = 0.025) and DIC (*r*^2^ = 0.5040, *P* = 0.032), while in the summer they were T (*r*^2^ = 0.4964, *P* = 0.030) and ORP (*r*^2^ = 0.4666, *P* = 0.036) (Figure 8). The main factors affecting the community structure of the *cbbM* gene-containing CO_2_-fixing bacteria in the summer were CA (*r*^2^ = 0.5401, *P* = 0.015) and DIC (*r*^2^ = 0.4891, *P* = 0.035) (Figure 9), whereas in the winter, there was no significant correlation between the environmental factors and the community structure. The distribution of various sites in the RDA diagram was scattered, indicating that the community structures of CO_2_-fixing bacteria at various sites varied due to the differences in environmental factors.

**FIGURE 10:**
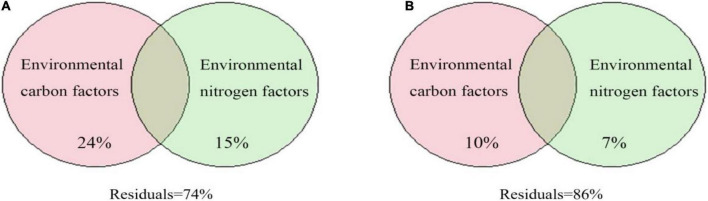
Variance partitioning analysis (VPA) of CO_2_-fixing bacterial communities containing the *cbbL* **(A)** or *cbbM* **(B)** genes based on environmental carbon and nitrogen factors.

The results of VPA showed that the environmental carbon and nitrogen factors explained 24 and 15% of the variation, respectively, in the *cbbL* gene-containing CO_2_-fixing bacterial community (Figure 10A). For the *cbbM* gene-containing CO_2_-fixing bacterial community, the environmental carbon and nitrogen factors explained 10 and 7% of the variation, respectively (Figure 10B). Thus, carbon and inorganic nitrogen compounds in the groundwater affected the variation in the CO_2_-fixing bacterial community.

**FIGURE 11:**
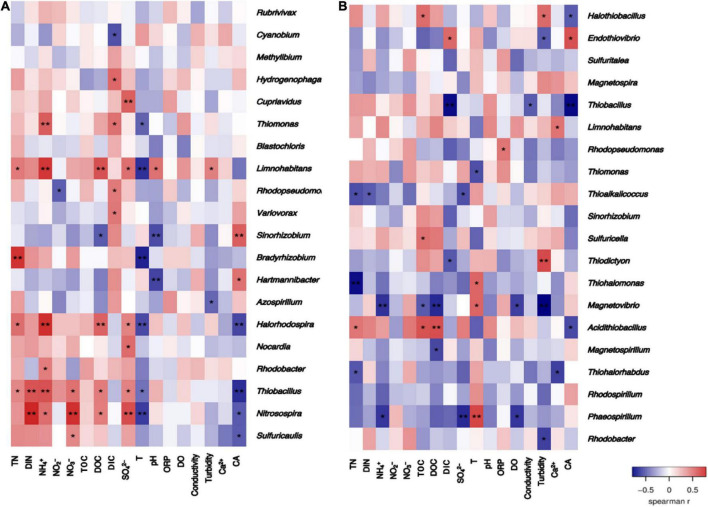
Heatmaps of the Spearman correlation between the distribution of the CO_2_-fixing bacterial communities containing the *cbbL* **(A)** or *cbbM* **(B)** genes and environmental factors in the Huixian karst groundwaters.

Spearman correlation heatmap analysis was conducted between the relative abundance of varied-fixing bacterial genera and the groundwater environmental factors (Figure 11). The relative abundance of more bacterial genera in the *cbbL*-gene containing bacterial community was significantly correlated with the concentration of inorganic nitrogen components in the groundwaters. Specifically, the relative abundances of *Thiobacillus*, *Halorhodospira*, *Limnohabitans*, *Thiomonas*, *Rhodobacter*, and *Nitrosopira* were significantly positively correlated with NH_4_^+^ (*P <* 0.05). *Rhodopseudomonas* was negatively correlated with NO_2_^–^ (*P <* 0.05). *Thiobacillus*, *Nitrosospira*, and *Sulfuricaulis* were significantly positively correlated with NO_3_^–^ (*P <* 0.05). *Thiobacillus* and *Nitrosospira* were significantly positively correlated with the concentration of dissolved inorganic nitrogen (DIN) (*P <* 0.05). For the *cbbM*-gene containing CO_2_-fixing bacterial community, there were fewer bacterial genera which were significantly correlated with the concentration of inorganic nitrogen compounds in the groundwaters. *Phaeospirillum* and *Magnetovibrio* were negatively correlated with NH_4_^+^ (*P <* 0.05). *Thioalkalicoccus* was negatively correlated with the concentration of inorganic nitrogen compounds (*P <* 0.05).

Other groundwater environmental factors were also significantly correlated with the relative abundance of many bacterial genera in the CO_2_-fixing bacterial community (*P <* 0.05). For example, DOC was significantly negatively correlated with *Sinorhizobium* (*P <* 0.05), and significantly positively correlated with *Limnohabitans*, *Halorhodospira*, *Thiobacillus*, and *Nitrosospira* (*P <* 0.05) in the *cbbL*-gene containing carbon-fixing bacterial community. For the *cbbM*-gene containing CO_2_-fixing bacterial community, DOC was significantly negatively correlated with *Magnetovibrio* and *Magnetospirillum* (*P <* 0.05), and significantly positively correlated with *Acidithiobacillus* (*P <* 0.05).

## Discussion

### Community structure and diversity of CO_2_-fixing bacteria in the Huixian karst groundwaters

The *cbbL* and *cbbM* genes were detected in all sampling sites of the Huixian karst groundwaters. The *cbbL* and *cbbM* CO_2_-fixing gene abundances were higher in the Huixian karst groundwaters than in freshwater lakes (Alfreider et al., 2017), seawaters (Fang et al., 2015), and pristine limestone aquifers (Herrmann et al., 2015). These results indicated that a large number of autotrophic CO_2_-fixing bacteria used the Calvin-Benson cycle for carbon fixation in the Huixian karst groundwaters. Autotrophic CO_2_-fixing bacterial communities play an important role in the CO_2_ fixation in aquatic environments (Zhao et al., 2020). The abundant CO_2_-fixing bacterial communities implied a higher carbon fixation potential in the Huixian karst groundwaters. In most samples from the Huixian karst groundwaters, the ratio of *cbbM*/16S rRNA was significantly higher than that of *cbbL*/16S rRNA, suggesting that the CO_2_-fixing bacterial communities using the RubisCO Type II for carbon assimilation accounted for a higher proportion in the Huixian karst groundwaters. Low O_2_ and high CO_2_ concentration are conducive to the growth of *cbbM* gene-containing CO_2_-fixing bacterial communities (Badger and Bek, 2008). Due to the special water-rock-gas interaction in the karst groundwaters, the concentration of CO_2_ dissolved in the water is relatively high, which may favor the growth of the *cbbM* gene-containing CO_2_-fixing bacterial community in this karst groundwater environment.

The diversity index of the CO_2_-fixing bacterial community containing the *cbbL* gene was higher than that containing the *cbbM* gene in most samples from the Huixian karst groundwaters. Thus, the diversity of CO_2_-fixing bacterial communities using RubisCO Type I for carbon assimilation was higher in the karst groundwaters. Microbial community diversity responds to changes in environmental physicochemical properties (Delgado-Baquerizo et al., 2016; Cavicchioli et al., 2019). A high level of microbial diversity is associated with ecosystem stability. In this study, DIN was significantly negatively correlated with the diversity index of the *cbbL* gene-containing bacterial community. The diversity index of *cbbM* gene-containing bacterial community was significantly negatively correlated with NH_4_^+^ and NO_2_^–^. These data indicated that nitrogen influx in the Huixian karst groundwaters decreased the diversity, compromising the stability of the CO_2_-fixing bacterial community.

The Proteobacteria and Cyanophyta were the main predominant phyla in the CO_2_-fixing bacterial community, and Proteobacteria occupied a high proportion in most sampling sites from the Huixian karst groundwaters. The Betaproteobacteria and Gammaproteobacteria of the phylum Proteobacteria were the dominant bacterial classes. These data were basically consistent with the reports (Ji et al., 2016) that the CO_2_-fixing bacterial communities in the surface water of northern South China Sea were dominated by Gamma- and Betaproteobacteria, Cyanobacteria, and Firmicutes.

*Rubrivivax* of the Betaproteobacteria was the dominant genus in the *cbbL* gene-containing CO_2_-fixing bacterial community in the Huixian karst groundwaters. Members in this genus can grow photoautotrophically under light conditions and chemoautotrophically under dark conditions, and they can be found in soils, lakes, and groundwaters (Kane et al., 2001; Gomila et al., 2010; Wu et al., 2015). Gao et al. (2019) reported that *Rubrivivax* was also associated with a denitrifying community. Zhao (2016) established that *Rubrivivax gelatinosus* was the main denitrifier in low ammonia-nitrogen water bodies and may play an important role in the removal of nitrogen pollution in water bodies. Members of *Hydrogenophaga* was also found in large numbers in the *cbbL* gene-containing CO_2_-fixing bacterial community in the present study. *Hydrogenophaga* belongs to hydrogen-oxidizing bacteria. As the chemoautotrophic bacteria with the fastest growth, hydrogen-oxidizing bacteria can obtain energy by oxidizing H_2_ and assimilate inorganic carbon source into organic carbon (Hu et al., 2009; Meng et al., 2010). Tan (2016) found that *Halothiobacillus*, *Sulfuritalea*, *Magnetospira*, *Thiobacillus*, *Limnohabitans*, and *Rhodopseudomonas* were the main autotrophic sulfur-oxidizers in Pearl River waters and possessed both carbon-fixing functional genes and sulfur-oxidizing functional genes. *Thiobacillus* and *Halothiobacillus* have been identified in the *cbbM* gene-containing CO_2_-fixing bacterial community in ammonia nitrogen-polluted groundwater (Alfreider et al., 2012). In the present study, a large number of both genera were also found in the *cbbM* gene-containing CO_2_-fixing bacterial community. *Halothiobacillus* accounted for a large proportion, and the highest relative abundance was 77.8%. *Halothiobacillus* is a type of chemoautotrophic sulfur-oxidizing bacteria, which carbon and energy sources required for growth and metabolism are completely derived from the fixation of CO_2_ and the oxidation of inorganic sulfur compounds (Shively et al., 1998; Kelly and Wood, 2000). Therefore, most *cbbM* gene-containing CO_2_-fixing bacterial communities in the Huixian karst groundwaters were related to sulfur oxidation. Herrmann et al. (2015) also found that a large fraction of bacteria in pristine limestone aquifers had the genetic potential for autotrophic CO_2_ fixation, with energy most likely provided by the oxidation of reduced sulfur and nitrogen compounds.

### Effects of environmental factors on CO_2_-fixing bacterial communities in the Huixian karst groundwaters

The VPA revealed that environmental carbon and nitrogen factors greatly influenced the CO_2_-fixing bacterial communities in in the Huixian karst groundwaters. Carbon and nitrogen compounds can serve as energy sources (Yang and Wu, 2020) as well as for assimilation into cell biomass. DIC is abundant as HCO_3_^–^ in the karst groundwaters (Table 2). Tang et al. (2018) found that DIC was positively correlated with the composition of the CO_2_-fixing bacterial community in lake surface sediments in the northern Qinghai-Tibetan Plateau, which was comparable to that of the present study. Extracellular CA catalyzes the conversion of HCO_3_^–^ into CO_2_, efficiently transporting CO_2_ into cells, and helps maintain high substrate concentration for the RubisCO enzyme active center. The CA activity in the groundwaters reflects the extracellular CA activity of autotrophic microbes to some extent. The correlation between the composition and abundance of CO_2_-fixing bacteria and the groundwater CA activity is a captivating paradigm. The CA results in the present study were comparable to the positive correlation between species and abundance of microalgae and the water CA activity previously reported for the Huixian karst wetland watershed (Yan et al., 2020).

The increase of inorganic nutrients, especially of nitrogen compounds, can enhance the number of CO_2_-fixing bacteria in aquatic ecosystems (Teufel et al., 2017). Yue et al. (2019) reported that increases of organic carbon and nitrogen compounds led to increased abundance of CO_2_-fixing genes in freshwater and hypersaline lakes of the Tibetan Plateau. In the present study, DIN was significantly positively correlated with the *cbbL* gene abundance, suggesting that the inflow of agricultural nitrogen into the Huixian karst groundwaters stimulated the growth of CO_2_-fixing bacterial communities in the groundwater system. In addition, Kamble et al. (2014) found that the addition of organic carbon significantly promoted bacterial growth in some high pH and high salinity soils. In the present study, the abundances of the *cbbL* and *cbbM* genes were significantly positively correlated with DOC, suggesting that the karst groundwaters with higher DOC content contained more CO_2_-fixing bacteria.

Additionally, the community structure of CO_2_-fixing bacteria in the Huixian karst groundwaters had significant correlation with groundwater T and ORP. It is well established that water temperature and nutrients affect the community structure of CO_2_-fixing bacteria in aquatic environments (Yue et al., 2019). Temperature controls the biological activity and thus affects the rate of microbial metabolism (Gillooly et al., 2001; Brown et al., 2004). Nutrient availability and suitable temperature are among the main drivers of the formation of microbial communities in aquatic ecosystems (Gilbert et al., 2009; Cross et al., 2015). Moreover, Alfreider et al. (2012) found that the differences in redox condition led to differences in the distribution and diversity of autotrophic carbon-fixing bacteria. These results were comparable to those of the present study.

### Differences in the distribution of CO_2_-fixing bacterial community in Huixian karst groundwaters with different nitrogen levels

This study compared the differences in the CO_2_-fixing bacterial community structure between high and low nitrogen levels through LEfSe analysis to further understand their key role in the nitrogen cycle process in the karst groundwaters. *Nitrosospira* and *Thiobacillus* (containing *cbbL* gene), and *Rhodobacter* (containing *cbbM* gene) were enriched in high nitrogen groundwaters. The Spearman correlation analysis showed that the relative abundances of *Nitrosospira* and *Thiobacillus* were significantly positively correlated with the concentrations of NH_4_^+^, NO_3_^–^ and DIN (*P* < 0.05). These results suggested that *Nitrosospira* and *Thiobacillus* responded positively to changes in nitrogen in the karst groundwaters, and may actively participate in the carbon and nitrogen cycles in the karst groundwater. Oved et al. (2001) found that *Nitrosospira*-like populations were dominant in soils irrigated with fertilizer-amended water. Shaw et al. (2006) reported that *Nitrosospira* spp. could use NO_2_^–^ to produce N_2_O via a nitrifier denitrification pathway. They suggested that this nitrifier-denitrification path may be a universal trait in ammonia-oxidizing bacteria of the Betaproteobacteria. Some *Thiobacillus* species are denitrifiers, such as *Thiobacillus denitrificans*, which is a sulfur-oxidizing, autotrophic denitrifier, and is one of the main species in the biological denitrification (Ye and Zuo, 2011). Some *Rhodobacter* species have been reported to be associated with the simultaneous nitrification and denitrification process (Moura et al., 2018). For nitrogen-polluted groundwater with oligoorganic carbon sources, autotrophic denitrification only requires inorganic carbon sources, thus avoiding the secondary pollution of water bodies by the addition of organic carbon sources, which is an effective and safe way to deal with nitrogen pollution (Capua et al., 2019). Autotrophic denitrifying bacteria have been applied in the removal of nitrogen pollution in groundwater systems. For example, Moon et al. (2008) removed nitrate via denitrification in groundwater samples by inoculating a bacterial consortium containing *Thiobacillus denitrificans* as a key member. The denitrifying community almost completely transformed nitrate (60 mg N L^–1^) to dinitrogen gas. The discovery of bacterial genera related to autotrophic denitrification in the Huixian karst groundwaters suggests that CO_2_-fixing bacteria are involved in nitrogen transformations and are important in the nitrogen removal in karst groundwater ecosystem.

## Conclusion

In the Huixian karst groundwaters, the abundance of the *cbbM* gene was higher than that of the *cbbL* gene. The phylum Proteobacteria was dominant in the autotrophic CO_2_-fixing bacterial communities containing the *cbbL* and *cbbM* genes. *Rubrivivax* and *Methylibium* were the dominant genera in the *cbbL* gene-containing CO_2_-fixing bacterial communities, while *Halothiobacillus* and *Endothiovibrio* were dominant genera in the *cbbM* gene-containing bacterial communities. In addition to environmental carbon factors (DIC, DOC, TOC, and CA), the abundance and diversity of autotrophic CO_2_-fixing bacterial communities containing the *cbbL* and *cbbM* genes in the karst groundwaters were modulated by inorganic nitrogen compounds. There were significant negative correlations between DIN, NH_4_^+^, NO_2_^–^ and the community diversity of autotrophic CO_2_-fixing bacteria. Thus, the inflow of nitrogen increased the abundance of autotrophic CO_2_-fixing bacteria but reduced the community diversity of autotrophs in the karst groundwaters. Some key autotrophic CO_2_-fixing bacterial genera such as *Nitrosospira* and *Thiobacillus* were enriched in the karst groundwaters with high nitrogen levels, and their respective roles in nitrification and denitrification affect nitrogen biotransformations in the karst groundwaters. These findings provide ongoing scientific basis for in-depth understanding of the microbial autotrophs and nitrogen cycling in karst groundwater systems, and provide an important reference for the protection and utilization of karst groundwater resources.

## Data availability statement

The datasets presented in this study can be found in online repositories. The names of the repository/repositories and accession number(s) can be found in the article.

## Author contributions

WL designed the experiments. XW, TS, and XP collected all samples. XW conducted the experiments and wrote the manuscript. XW and WL analyzed the data. WL, AC, YX, MZ, and LY revised the manuscript. All authors contributed to the article and approved the submitted version.

## Footnotes

1
http://huttenhower.sph.harvard.edu/galaxy/
