# Supplementary material for: Community characteristics of autotrophic CO2-fixing bacteria in karst wetland groundwaters with different nitrogen levels
Source: Front Microbiol. 2022 Aug 15;13:949208. doi: 10.3389/fmicb.2022.949208 (PMC9421164; doi:10.3389/fmicb.2022.949208)
Supplement: Supplementary file 1 [file Data_Sheet_1.PDF]

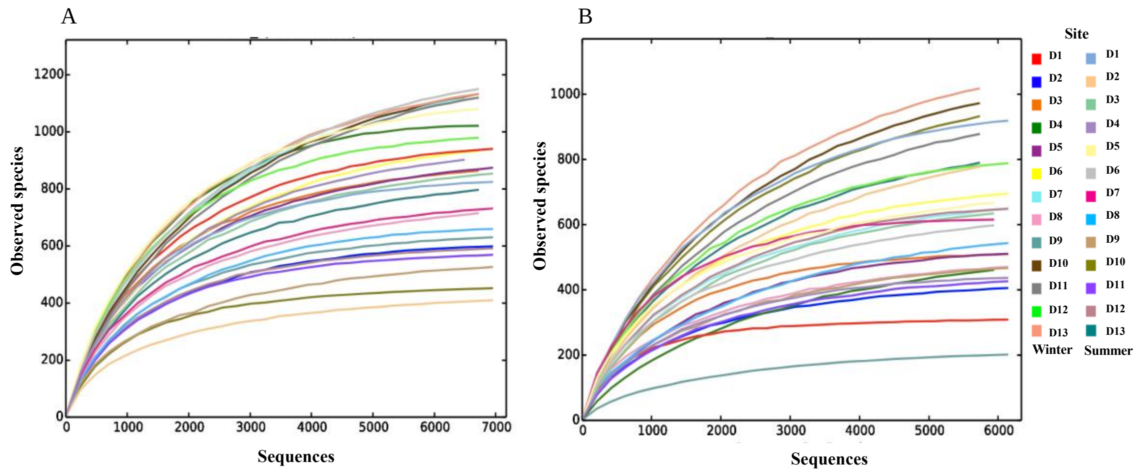

Fig. S1. Rarefaction curves of carbon-fixing genes in the Huixian karst groundwaters. A: *cbbL* gene; B: *cbbM* gene.

Table S1 Amplification primers of 16S rRNA and carbon-fixing functional genes.

Gene name	Primer number	Base sequence (5'-3')	Reference
16S rRNA	1055f	ATGGCTGTCGTCAGCT	Ferris et al., 1996
	1392r	ACGGGCGGTGTGTAC	
cbbL	*cbbL*-f	CGGCACSTGGACCACSGTSTGGAC	Alfreider et al., 2003
	cbbL-r	GTARTCGTGCATGATGATSGG	
cbbM	*cbbM*-f	GGCACCATCATCAAGCCCAAG	Alfreider et al., 2003
	cbbM-r	TCTTGCCGTAGCCCATGGTGC	

References

Alfreider, A., Vogt, C., Hoffmann, D., Babel, W., 2003. Diversity of ribulose-1,5-bisphosphate carboxylase/oxygenase large-subunit genes from groundwater and aquifer microorganisms. Microb. Ecol. 45(4), 317-328.

Ferris, M.J., Muyzer, G., Ward, D.M.,1996. Denaturing gradient gel electrophoresis profiles of 16S rRNA-defined populations inhabiting a hot spring microbial mat community. Appl. Environ. Microbiol. 62(2), 340-346.

Table S2 Sequence number, OTU number and diversity index of *cbbL* gene-containing carbon-fixing bacterial community in the Huixian karst groundwaters.

Season	Site	No. of sequences	No. of OTUs	Simpson	Shannon	Chao1	ACE
Winter	D1	46991	4853	0.985667	8.55	969.78	977.72
	D2	41829	3047	0.982047	7.53	604.54	614.01
	D3	32799	4575	0.987807	8.46	954.91	924.96
	D4	27067	5362	0.992412	8.94	1021	1021
	D5	39358	4623	0.991459	8.43	933.2	941.81
	D6	38404	4961	0.98228	8.18	975.47	1010.14
	D7	34863	5923	0.991521	8.79	1249.01	1280.22
	D8	40602	3828	0.97848	7.68	833.83	793.69
	D9	41413	3400	0.956686	7.21	655.49	657.99
	D10	48116	6092	0.988824	8.68	1216.02	1282.12
	D11	44927	5991	0.984068	8.46	1213.25	1285.71
	D12	46645	5174	0.995845	9.03	1006.05	1013.02
	D13	44661	6016	0.992252	8.96	1226.49	1250.65
Summer	D1	47860	4230	0.992457	8.48	851.64	852.48
	D2	55482	2209	0.885422	5.91	434.92	444.33
	D3	48917	4545	0.989011	8.28	908.8	915.1
	D4	43018	5059	0.989692	8.4	955.03	972.61
	D5	46734	5677	0.995693	9.15	1140.88	1143.6
	D6	75584	6102	0.994182	8.93	1228	1295.08
	D7	29263	3721	0.991659	8.18	761.02	780.82
	D8	47325	3320	0.98278	7.64	667.84	677.79
	D9	49503	2641	0.982774	7.34	552.45	571.14
	D10	27841	2344	0.924961	6.46	478.74	469.29
	D11	33674	3219	0.975582	7.4	592.61	586.25
	D12	48771	3034	0.970089	7.3	614.64	613.1
	D13	30203	4492	0.982832	7.98	903.76	879.6

Table S3 Sequence number, OTU number and diversity index of *cbbM* gene-containing carbon-fixing bacterial community in the Huixian karst groundwaters.

Season	Site	No. of sequences	No. of OTUs	Simpson	Shannon	Chao1	ACE
Winter	D1	53458	1842	0.973292	6.68	322.16	315.57
	D2	47326	2428	0.951305	6.12	440.6	438.88
	D3	38569	2973	0.945186	6.64	521.44	521.82
	D4	35990	2744	0.62869	3.86	531.5	572.04
	D5	34286	3064	0.753742	4.97	525.8	539.47
	D6	41624	4126	0.982455	7.71	729.7	748.37
	D7	36638	3868	0.97698	7.37	701.74	721.65
	D8	47410	2763	0.973257	6.95	495.18	517.25
	D9	50900	1203	0.523732	2.64	209.8	220.38
	D10	35532	5803	0.971748	7.87	1076.78	1162.87
	D11	37676	5243	0.968752	7.56	974.7	1026.77
	D12	59556	4572	0.984204	7.87	805.55	832.65
	D13	35777	6104	0.971256	8.01	1119.28	1189.85
Summer	D1	57775	5505	0.978433	8.14	942.61	974.6
	D2	63040	4652	0.93927	7.11	927.43	993.82
	D3	28263	3790	0.883366	6.2	685.72	706.73
	D4	36843	2616	0.923316	5.99	441.97	450.59
	D5	33256	3972	0.98866	7.91	758.3	760.95
	D6	49929	3546	0.963013	7.06	686.4	684.62
	D7	38261	3691	0.993278	8.26	616	616
	D8	57073	3215	0.984337	7.11	577.68	609.12
	D9	63411	2802	0.986582	7.12	486.25	512.97
	D10	39273	5580	0.962821	7.64	1089.11	1120.09
	D11	37691	2509	0.915221	5.77	447.13	460.4
	D12	57235	3886	0.97701	7.31	656.91	674.04
	D13	37776	4703	0.95103	7.4	882.73	915.59
